# STEME: A Robust, Accurate Motif Finder for Large Data Sets

**DOI:** 10.1371/journal.pone.0090735

**Published:** 2014-03-13

**Authors:** John E. Reid, Lorenz Wernisch

**Affiliations:** MRC Biostatistics Unit, Institute of Public Health, Cambridge, United Kingdom; King's College London, United Kingdom

## Abstract

Motif finding is a difficult problem that has been studied for over 20 years. Some older popular motif finders are not suitable for analysis of the large data sets generated by next-generation sequencing. We recently published an efficient approximation (STEME) to the EM algorithm that is at the core of many motif finders such as MEME. This approximation allows the EM algorithm to be applied to large data sets. In this work we describe several efficient extensions to STEME that are based on the MEME algorithm. Together with the original STEME EM approximation, these extensions make STEME a fully-fledged motif finder with similar properties to MEME. We discuss the difficulty of objectively comparing motif finders. We show that STEME performs comparably to existing prominent discriminative motif finders, DREME and Trawler, on 13 sets of transcription factor binding data in mouse ES cells. We demonstrate the ability of STEME to find long degenerate motifs which these discriminative motif finders do not find. As part of our method, we extend an earlier method due to Nagarajan et al. for the efficient calculation of motif E-values. STEME's source code is available under an open source license and STEME is available via a web interface.

## Introduction

### Transcriptional regulation

Spatio-temporal regulation of gene expression is critical for the correct function of many cellular processes. There are several mechanisms through which the genome achieves this control. Transcriptional regulation is one of the most prevalent and highly studied mechanisms. In transcriptional regulation, proteins called transcription factors (TFs) bind to DNA and influence the rate of transcription of particular genes. These TFs usually exhibit sequence specific binding specificities such that they preferentially bind to particular binding sites in the genome (TFBSs).

Several high-throughput experimental techniques have recently been developed to investigate the locations at which TFs bind. These include ChIP-chip [1]–[4], ChIP-seq [5], [6], and DamID [7]. A typical experiment will report that a given TF binds to thousands of regions across the genome under a particular condition. These techniques cannot determine the exact location of the TFBSs: the regions they report can be several hundred base pairs long.

Given the binding data from one or several of these experiments it is natural to ask if we can identify the sequence binding preferences of the TFs. With this information we can determine the exact location of the TFBSs which can be useful to investigate interactions between TFs. The sequence preferences also allow us to computationally predict binding sites under conditions for which we do not have experimental data. The task of determining the sequence preferences of a TF from binding data is termed motif finding.

### Motif finders

The sequence binding preferences of TFs can be modelled in several ways, such models are called *motifs*. The simplest motif model is the consensus sequence which defines the preferred base in each position of the TFBS. This can be extended to model some variability by replacing bases with characters from the IUPAC nucleotide code. The most general model in which the positions in the TFBS are independent is the position weight matrix (PWM). The PWM models the probability of every base at each position in the TFBS. The PWM remains the most popular and flexible motif model.

In general, motif finders can be classified as probabilistic or enumerative. Probabilistic motif finders optimise or learn the parameters of a generative model, whereas enumerative motif finders search in the space of consensus sequences for words that optimise some suitable statistic. MEME [8], AlignAce [9] and HMS [10] are examples of probabilistic motif finders. DREME [11] and Trawler [12] are examples of enumerative motif finders. The two most popular methods for learning the parameters of models in probabilistic motif finders are the Expectation-Maximisation (EM) algorithm and Gibbs sampling [13]. In a previous publication [14] we showed how suffix trees could be used to implement an efficient approximation to the EM algorithm that is at the core of MEME. The main contribution of this work is to show how to use suffix trees to efficiently implement the rest of the MEME algorithm.

MEME [8] was one of the first motif finders and is still one of the most popular. It performed well in a benchmark evaluation by Tompa et al. [15]. MEME uses the EM algorithm to iteratively refine estimates of PWMs. However, the EM algorithm only converges to a local optimum. This forces MEME to try many different initialisations when looking for the global optimum. The search for the best initialisations is inefficient and makes MEME impractical to run on large data sets. Our motif finding method, STEME, uses the EM algorithm in a similar manner as MEME. However, it incorporates features designed to make it applicable to much larger data sets.

Recently much research has been done on discriminative motif finders [11], [12], [16]–[20]. Motif finders such as MEME use a generative model that typically explains the input sequences in terms of a background model and a model for binding sites. They learn motif representations that optimise some significance statistic. This statistic is usually based on the strength of the motif and the number of binding sites in the input sequences. In contrast, discriminative motif finders find motifs that distinguish the input sequences from an additional set of control sequences. Typically some statistic that takes into account how many input and control sequences have binding sites is optimised. In this way discriminative motif finders use information from the control sequences as well as the input sequences. Discriminative motif finders such as DREME [11] and Trawler [12] have become the methods of choice when a suitable set of control sequences are available. In this work we compare the performance of STEME to DREME and Trawler.

Motif finding algorithms appear to be caught in the ‘self-assessment trap’ as identified by Norel et al. [21], where the editorial policy of scientific journals dictates that investigators must evaluate their method against other methods. Norel et al. point out that in these evaluations, the novel method is reported as best an unreasonably high number of times. They argue that this bias is typically a result of a selective evaluation in the niche in which the algorithm performs best. Not wishing to fall into this trap, we have not tailored our evaluations to present STEME as a superior motif finding algorithm. We chose to evaluate STEME against other motif finders on 13 data sets that these motif finders had already been evaluated on.

In the rest of this paper we discuss the difficulty of evaluating motif finders; we describe the results of evaluating STEME, DREME and Trawler on 13 data sets from mouse ES cells; we show that STEME is better at finding long degenerate motifs in these data sets; we discuss how DREME and Trawler are sensitive to the choice of control sequences and how STEME is robust to this choice. Finally we present STEME's algorithm which includes a generalisation of a method by Nagarajan et al. [22] for calculating a motif's significance.

## Results and Discussion

### Difficulty of evaluating motif finders

Like many other tasks in computational biology such as protein interaction prediction [23] and gene regulatory network inference [24], the lack of a gold standard makes the evaluation of motif finding algorithms difficult at best. The results of motif finding algorithms are usually scored against matches to known motifs from the literature. Any difference between the literature motifs and the binding specificities of the factor will bias the evaluation. This can happen in several ways: *in vitro* derived motifs in the literature may not accurately represent *in vivo* specificities; the transcription factors may have context dependent binding specificities subject to the presence of co-factors [25]; or a factor may have more than one mode of binding [26]. Comparison of motif finders in this way is also difficult. Presumably a motif finder that reports one matching motif is better in some way than one that reports 100 motifs of which one matches. This distinction can be difficult to quantify. However, the volume of data from high-throughput experiments like ChIP-chip, ChIP-seq, and DamID provides opportunities for the empirical evaluation of motif finding algorithms.

### Evaluation on ChIP-seq data from Chen et al

In order to evaluate STEME, we used ChIP-seq data from mouse ES cells [27] for 13 sequence-specific TFs (Nanog, Oct4, STAT3, Smad1, Sox2, Zfx, c-Myc, n-Myc, Klf4, Esrrb, Tcfcp2l1, E2f1, and CTCF). These data have been well used as test data for motif finders and this allows us to compare the the results of our evaluation against those of others. Indeed, the author of DREME used the same data to evaluate his method [11]. We evaluated three motif finders: STEME, DREME and Trawler. The evaluation was configured as a discriminative task where the methods were asked to find motifs in the input sequences as compared to a set of control sequences. The authors of DREME and Trawler differ in their recommendations for control sequences: the DREME author recommends dinucleotide-shuffled versions of the input sequences and the Trawler authors recommend using 5 kb upstream promoter regions of genes. We used both types of control sequences in our evaluations.

For the analysis of the results we followed the DREME protocol. We used the TOMTOM tool [28] from the MEME suite to compare the discovered motifs to established motifs for the ChIP'ed transcription factor. We used motifs from the JASPAR core vertebrata database [29], mouse motifs from the UniProbe database [30] and individual motifs for Nanog [31], [32] and Smad1 [33] that were not in either of the JASPAR or UniProbe motif sets.

We show the results using the shuffled control sequences in Table 1 and the results using the promoter control sequences in Table 2. As expected, DREME and Trawler performed best when used with the control sequences recommended by their authors. DREME finds the correct motif in 10 out of the 13 data sets using the dinucleotide-shuffled control sequences but also reports over 300 motifs in total. STEME also finds 10 correct motifs but only reports 29 in total. Trawler performs best using the promoter control sequences, finding 10 of the correct motifs. The correct motif was never found on two of the data sets: Smad1 and E2f1 and Trawler was the only method to find the Zfx motif.

**Table 1:** Results using the dinucleotide-shuffled control sequences.

	DREME	Trawler	STEME
Nanog	1/34	4/7	1/2
Oct4	1/13	8/8	1/2
STAT3	1/10	1/4	1/3
Smad1	-/6	-/6	-/3
Sox2	1/16	14/18	1/2
Zfx	-/23	-/8	-/3
c-Myc	1/11	-/1	1/2
n-Myc	1/24	1/5	1/3
Klf4	1/28	1/3	1/2
Esrrb	1/32	1/5	1/3
Tcfcp2l1	1/43	-/1	1/2
E2f1	-/26	-/5	-/1
CTCF	1/36	-/2	1/1
***Total***	10/302	7/73	10/29

For each of the three methods, we show the rank of the most significant motif that matched a known motif for the ChIP'ed factor and how many motifs were reported in total. The last row shows how many times a correct motif was reported against how many motifs were reported in total.

**Table 2:** Results using the promoter control sequences.

	DREME	Trawler	STEME
Nanog	1/29	4/4	1/2
Oct4	3/15	2/3	1/1
STAT3	3/12	1/1	1/2
Smad1	-/6	-/3	-/2
Sox2	1/16	1/2	1/2
Zfx	-/10	1/2	-/-
c-Myc	-/9	1/2	-/-
n-Myc	-/8	-/1	-/-
Klf4	3/14	1/1	1/1
Esrrb	1/30	1/1	1/1
Tcfcp2l1	-/36	1/22	1/1
E2f1	-/15	-/1	-/-
CTCF	1/44	1/8	1/1
***Total***	7/244	10/51	8/13

For each of the three methods, we show the rank of the most significant motif that matched a known motif for the ChIP'ed factor and how many motifs were reported in total. The last row shows how many times a correct motif was reported against how many motifs were reported in total.

### STEME is better than Trawler and DREME at finding longer degenerate motifs

Discriminative methods such as DREME and Trawler may be quicker than STEME but they search in the space of regular expressions for motifs. The size of this space grows exponentially as the width of the motif increases making it difficult to find wide motifs. STEME's approach, like MEME's, differs in that it searches in the space of suitable starting seeds and uses the EM algorithm to move from these seeds to motifs. This enables STEME to reach a bigger space of putative motifs including longer and more degenerate motifs. We found that DREME and Trawler were excellent at finding short motifs that matched part of long literature motifs but they did not recover them in their entirety. However, STEME was able to recover these motifs. We show the two most extreme examples here.

**Figure 1:**
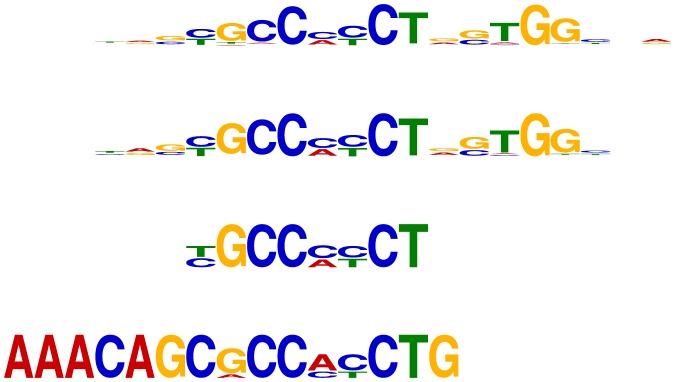
Logos for CTCF motifs. First: The reverse complement of the known CTCF motif from the JASPAR database (MA0139.1). Second: The motif recovered by STEME using the shuffled control sequences. STEME also recovers a near identical motif using the promoter control sequences. Third: The reverse-complement of the motif recovered by DREME using the shuffled control sequences. Fourth: One of the CTCF-like motifs recovered by Trawler using the promoter control sequences.

**Figure 2:**
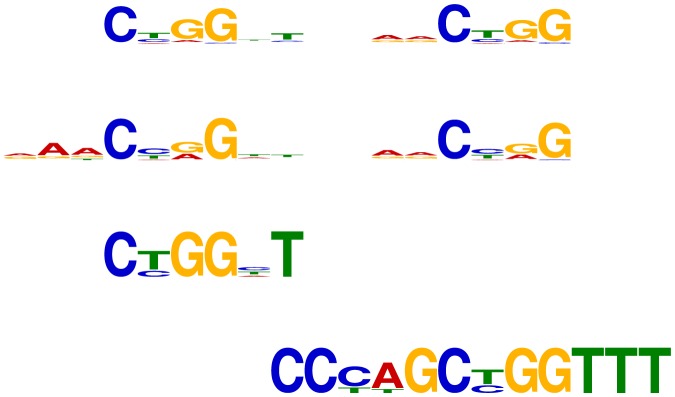
Logos for Tcfcp2l1 motifs. First: The reverse complement of the known Tcfcp2l1 motif from the JASPAR database (MA0145.1). Second: The motif recovered by STEME using the shuffled control sequences. STEME recovered a near identical motif using the promoter control sequences. Third: The reverse-complement of the motif recovered by DREME using the shuffled control sequences. Fourth: The Tcfcp2l1 motif recovered by Trawler when using the promoter control sequences.

First, the CTCF motif reported by DREME using the shuffled control sequences is a good match to part of the known CTCF motif. On the other hand, STEME recovers an almost exact match to the entire motif (see Figure 1). Using the promoter control sequences, Trawler recovers part of the CTCF motif. The TOMTOM E-value for STEME's match to the CTCF motif is 9.7e-14, as compared to 3.4e-7 for DREME's match, demonstrating that the STEME motif is much closer to the known motif. Second, the Tcfcp2l1 motif that STEME recovers matches the full length motif of Tcfcp2l1 binding as a homodimer. DREME's and Trawler's motifs (with the shuffled and promoter control sequences respectively) represent the binding specificity of one half of this homodimer and are not as close a match to the reference motif from the JASPAR database (see Figure 2). TOMTOM reports E-values of 1e-9 (respectively 1.7e-3) for STEME's (respectively DREME's) match to the known motif. For both TFs, STEME reports a good match to the full motif using both sets of control sequences. In contrast Trawler and DREME can only match part of the reference motif with one set of control sequences and do not always find a match at all with the other control sequences.

The ability to discover these longer degenerate motifs is the reason why Bailey argues that MEME is complementary to DREME: ‘Consequently, our algorithm complements rather than replaces existing motif discovery tools for the analysis of ChIP-seq data’ [11]. STEME is a motif finder with similar properties to MEME in this respect but unlike MEME it does not take a prohibitively long time to run on large data sets. MEME's authors suggest that sequences should be discarded when running MEME on large data sets. In practice this approach seems to work well as evidenced by the evaluation of MEME on the same data sets by Bailey [11]. We believe STEME has an advantage at least in a theoretical sense, in that it uses all of the data to infer the motifs.

### Running times

**Figure 3:**
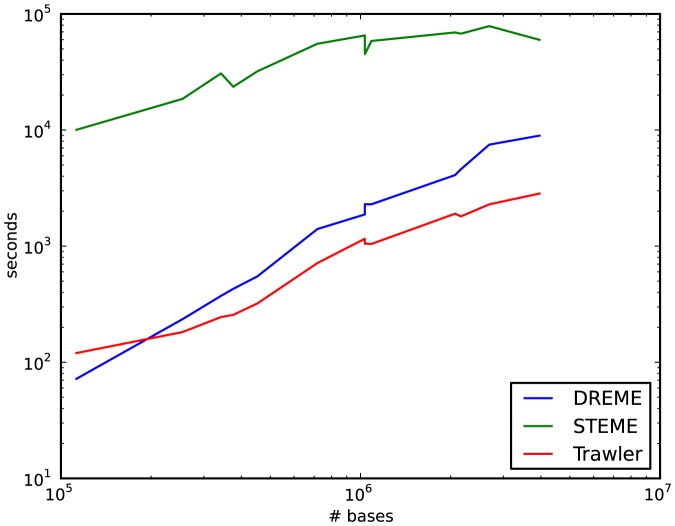
Log-log plot of timings for the three evaluated algorithms. STEME is slower than DREME and Trawler but this difference is less pronounced on larger data sets.

In Figure 3 we show the running times for the three algorithms on the data from Chen et al. STEME is up to two orders of magnitude slower than DREME and Trawler on the smaller data sets. On larger data sets this is reduced to roughly one order of magnitude. STEME has a configurable run time in that the user can specify how long it spends searching for good seeds to run the EM algorithm on. For our evaluations we used a fairly conservative value for this parameter of two hours. We note that it is not practical to run MEME, upon which STEME is modelled, on the larger data sets.

### The choice of control sequences is important

In our evaluation, there was a marked difference in performance between DREME and Trawler when different control sequences were used. DREME was more successful on dinucleotide-shuffled control sequences and Trawler was more successful with random promoter sequences. STEME showed a robust performance when using both sets of control sequences, finding the correct motif 10/13 times on the dinucleotide-shuffled control sequences compared to DREME which found the correct motif 10/13 times. On the promoter control sequences, STEME found the correct motif 8/13 times, compared to Trawler which found the correct motif 10/13 times.

Since dinucleotide-shuffled sequences are always available, why not exclusively use DREME with these as controls? When using shuffled sequences as controls, DREME reports large numbers of significant motifs: in our evaluation DREME reported an average of over 20 motifs per data set. Some of these motifs are very likely to be co-factors of interest to the experimenter. However, others may well be motifs for ubiquitous transcription factors that are also prevalent in other areas of the genome. These will be false starts for the experimenter who is typically interested in those factors that drive expression in the specific regions of the genome he has sequenced. Genomic sequences can show structure at Markov orders higher than one [34]. This structure is removed by the shuffling, hence some of these motifs may in fact just model this higher-order structure which may not be related to transcription factor binding. Whilst using dinucleotide-shuffled control sequences has been shown empirically to be a practical method of finding motifs, we believe that it is not ideal and if they are available, real genomic control sequences should be used additionally or instead of them.

DREME is sensitive to the choice of control sequences: using the promoter control sequences, DREME only reported 7 correct motifs across all 13 data sets. We could have relaxed DREME's significance threshold for reporting motifs but without any guidance for choosing a suitable threshold in this instance, we chose to report the results as they stand.

Bailey chose to use dinucleotide-shuffled sequences for the control set when evaluating DREME against Trawler [11]. It is possible this did not do Trawler justice.

### STEME as a discriminative motif finder

STEME is not a discriminative motif finder in that it does not explicitly optimise a statistic that discriminates between the input sequences and the control sequences. However, it does incorporate information from the control sequences when they are used to train its background Markov model. STEME's background model is an important part of its generative model and is used at every stage of its algorithm. 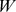-mers that are favoured by the background model will be less likely to modelled as binding sites. In this way, the background model can play a discriminative role in how STEME finds motifs.

This is not a novel approach: NestedMICA [35] uses a mosaic Markov model for the background sequence. NestedMICA's authors show that their rich background model can capture information present in the control sequences and make motif finding more sensitive. They recommend using a four class first order mosaic Markov model for mammalian sequences. STEME does not implement a full mosaic background model but our experience shows that using higher order Markov models can be beneficial. In particular, we have had successes with background Markov models of orders five and six (data not shown). Thijs et al. showed that fourth order models can be be superior to lower order models [36]. Their study was performed on a limited amount of sequence data which constrained it from investigating higher order models.

### Conclusions

Motif finding is a heavily studied area of computational biology and there are many excellent motif finders, each of which has demonstrated its superiority over some of its competitors. In this work we do not attempt to prove that STEME is superior to all these motif finders, indeed we would be surprised if it were. We compare STEME to two popular motif finders DREME and Trawler and showed that STEME predicts a comparable number of correct motifs. Unlike DREME and to some extent Trawler, STEME does not overwhelm the user with many motif predictions.

We also show how STEME can find longer more degenerate motifs than those found by the enumerative methods in the comparison. The results from TOMTOM show that these are closer matches to the known motifs from the literature.

We present several extensions to the original STEME EM approximation. These extensions enable STEME to be used as a fully-fledged motif finder in its own right. We think that these algorithms are of some theoretical interest. We have showed how to use suffix trees to implement approximations to the parts of the MEME algorithm our original publication did not address. In addition we made a small generalisation to a method for accurately computing 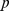-values due to Nagarajan et al.

Our analysis of the mouse ES binding data highlights some of the problems inherent in the comparison of motif finders. Many evaluations of motif finders only use simple statistics such as the number of correct motifs reported. We have shown that these do not always tell the whole story. Some motif finders report many significant motifs. These can be difficult for the user to interpret. Also, the quality of the match to the known motif is often ignored. Many evaluations score short motifs that match part of the known motif as highly as almost exact matches to the whole motif. A consensus as to the best way to compare motif finders would ensure motif finders are compared on an equal footing. This would help reduce the effects of the ‘self-assessment trap’ which we believe is prevalent in this and several other areas of computational biology.

## Materials and Methods

### Availability

STEME is available through a web interface (http://sysbio.mrc-bsu.cam.ac.uk/STEME/) where the user can upload their sequences and vary some of the basic STEME parameters. For more control over the full parameter set, STEME can be installed as a python package from PyPI (https://pypi.python.org/pypi/STEME/) and the source code is available on github (https://github.com/JohnReid/STEME).

### The STEME motif finding algorithm

The STEME algorithm in its previously described form [14] was an approximation to the EM algorithm for the type of mixture model used by motif finders such as MEME [8]. Here we describe how we have extended it into a fully-fledged motif finder. There are 3 major parts to these extensions. Firstly, we have extended MEME's model to allow for position-specific priors. Secondly, we have designed and implemented an algorithm that efficiently searches for suitable seeds with which to initialise EM. Lastly, we also present a generalisation of a previous algorithm by Nagarajan et al. for the significance calculations.

#### The model

STEME can search for motifs of different widths, however its probabilistic model only considers one width at a time. STEME's model is an extension of the model used by MEME: for a fixed width, 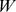, STEME models each 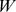-mer as an independent draw from a two-component mixture. One mixture component models binding sites, the other component models background sequence. We index the set of 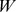-mers by 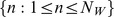 where 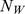 is the number of 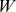-mers in the input sequences. We define 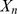 as the 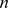
th 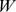-mer in the input sequences. We define a corresponding set of latent variables, 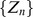, that represent whether each 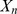 is drawn from the binding site component or the background component. We make an assumption that the 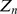 are independent. This is a simplifying assumption as the 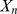 overlap. STEME uses the same technique as MEME to avoid problems resulting from this simplification. Without these techniques STEME tends to converge on motifs of low-complexity with self-overlapping binding sites. We discuss the techniques further below.

**Figure 4:**
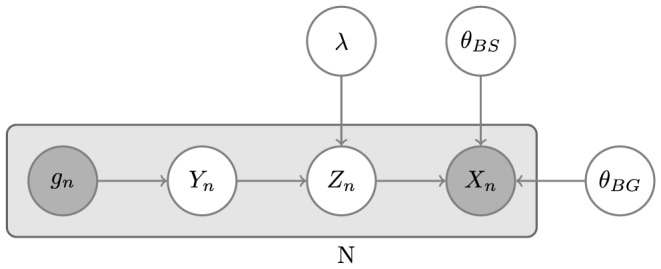
Our model in plate notation.

STEME extends MEME's mixture model to include position-specific priors. Position-specific priors were introduced by Narlikar et al. [37] and are a method by which almost any information that is position-specific can be introduced into the motif finding process. Typical examples of such information are: phylogenetic conservation [38]; nucleosome occupancy [37]; a negative set of control sequences (a.k.a. discriminative motif finding) [39]. In this work, we introduce two new variables into STEME's model: 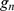 represents the prior probability that the 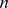
'th 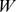-mer is not available to be a binding site; 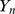 is an indicator variable that determines if 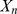 is available as a binding site. 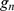 is observed data in our model and 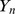 is a hidden variable. Note that 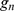 is parameterised in a negative sense. Typically most 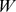-mers are available as binding sites, so in this parameterisation many 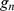 are 0. This allows STEME to use efficient sparse data structures to represent the few nonzero 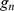 representing sites that are known to be unavailable. The model is depicted in Figure 4 and the model parameters are described in Table 3.

**Table 3:** The parameters of our model.

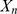	:	The 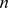 'th W-mer
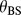	:	The parameters of the binding site model
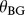	:	The parameters of the background model
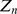	:	Indicator variable: determines whether 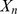 is a binding site
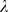	:	Prior probability for 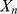 being a binding site
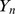	:	Indicator variable: determines if 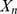 is available as a binding site
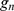	:	Position-specific prior on 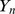

So we have
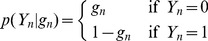
(1)If 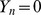 then the 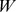-mer, 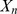, is not available as a binding site and 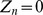


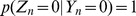
and if 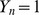 then 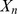 is available and 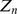 will be 1 according to the prior probability of a binding site, 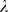


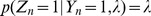
Now if 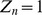, 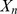 is drawn from the binding site model
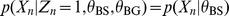
and conversely if 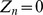, 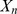 is drawn from the background model
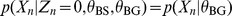
Summing out 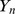


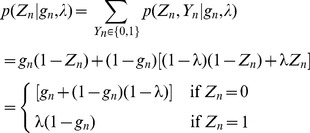
(2)giving the following likelihood
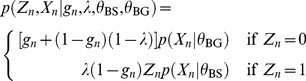
(3)and posterior
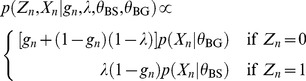
(4)The expectation of 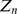 is
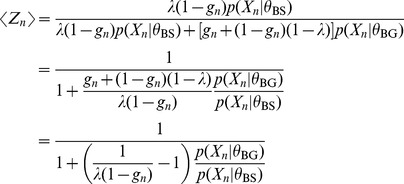
(5)


#### Branch-and-bound approximation

Several parts of the STEME algorithm enumerate the 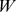-mers efficiently using a branch-and-bound descent of a suffix tree. These include searching for starts, the EM algorithm and finding instances of a motif. The efficiency of these descents depends on the availability of an upper bound for the 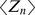. In the model presented in the original STEME algorithm [14], this bound was easy to calculate using an upper bound on 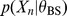 and a lower bound on 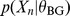. The introduction of position-specific priors means that we also need a lower bound on the 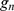 for each node in the suffix tree.

Each node in a suffix tree represents a common prefix of a set of suffixes. In our model each node 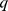 represents a common prefix 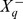 of a set of binding sites indexed by 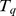


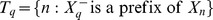
(6)For each node 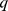 in the suffix tree we define bounds on the likelihoods of the binding sites indexed by 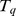 and on the position-specific priors associated with those binding sites 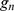


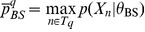
(7)

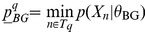
(8)

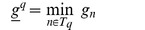
(9)Now we have the required bound on the expectation of 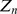 for all the binding sites prefixed at node 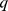 (c.f. Equation (5))
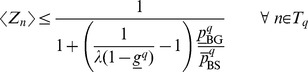
(10)The 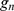 are fixed across all parts of the algorithm so we can calculate lower bounds 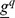 for all nodes 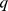 in the suffix tree as a pre-processing step. We recalculate these bounds when searching for multiple motifs.

Note that Equation (10) is an improvement over the bound given in the EM-algorithm of our original publication [14]. In that publication we used a lower bound of 0 for 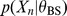 for part of the calculation. Here we have removed the need for this coarse bound.

Each part of the STEME algorithm that uses this branch-and-bound descent provides a global bound 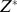. On each descent, we can ignore all the 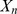 for which 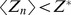. The bound on the likelihood under the PWM model that we can use at node 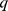 is given by
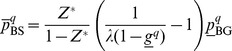
(11)


#### The binding site and background model

For the binding site component of the model STEME uses the popular position weight matrix (PWM) model where each position is treated independently.
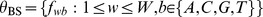
where 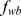 is the frequency of base 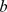 at position 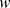 in the PWM. Other more complex binding site models are possible within the framework.


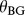
 is treated as constant throughout the STEME algorithm. A Markov model of an order specified by the user is built from the input sequences (or the control sequences if available). This model is used to calculate 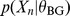 as a pre-processing step. The STEME algorithm could be easily extended to use estimates of the 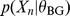 provided by the user, thereby enabling the use of custom background models.

#### Finding starting points

The EM algorithm finds a local maximum of the expected likelihood of the model. It is a proven technique for motif finding but when the EM algorithm is initialised with different starting points, different local maxima are often found. In order to improve their chances of finding a global maximum, motif finding algorithms such as MEME devote many of their resources to finding good starting points. STEME follows this design pattern.

The EM algorithm needs a consensus sequence and a number of sites to be initialised. Given a consensus sequence, 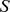, STEME initialises the binding site model, 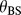, as
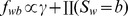
where 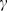 is a pseudo-count parameter and 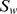 is the 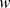
'th base of 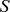. The number of sites is used to initialise the prior probability of a binding site
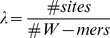
In general, the motif width and number of sites are not known ahead of time so STEME considers a range of values for both. These can be specified by the user but default to widths of 6, 8, 11 and 14 base pairs and numbers of sites between 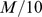 and 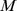, where 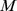 is the number of input sequences. STEME uses a geometrical progression to select values in the range specified. We denote the range of widths by 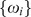 and the numbers of sites by 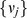 where 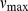 is the largest number of sites considered. For each combination of 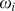 and 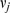 STEME searches for the best consensus sequences with which to initialise EM.

Given a potential starting point, 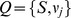, consisting of a consensus sequence, 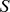, of width 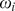 and a number of sites, 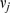, STEME uses a similar algorithm to MEME to calculate 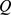
's score. STEME initialises its binding model with the consensus sequence, 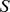. Using this model, STEME finds the best 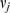 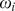-mers in the sense that they are the 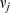 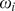-mers with the highest 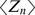. A PWM is built from these 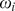-mers and the significance of its log-likelihood ratio is used as the score for 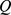. The significance calculation is described below.

For each width, 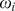, STEME's default behaviour is to consider every 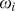-mer, 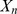, in the data as a consensus sequence and examine every starting point 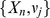 for all 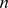 and 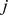. However, this can be very expensive when 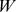 is large and the input sequences are numerous or long. STEME allows the user to limit the amount of time spent looking for starts. STEME divides this time equally amongst all possible of widths of motif. When looking for the starts for each width, STEME estimates how long it takes to evaluate each start and skips an appropriate number of subsequent starts so it finishes in the specified time.

When finding multiple motifs, MEME performs a search for starts before learning each motif. This is an expensive operation. STEME chooses to run this search for starts just once. It finds many starts for each 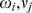 combination prior to looking for any motif. STEME does not just store the highest scoring starting points. Oftentimes the best starting points represent the same motif, only with minor edits in the 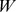-mer. STEME will only store starting points when the set of their best 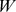-mers do not overlap any better starting point's 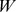-mers by 50% or more at a base pair level. In this manner, STEME avoids storing redundant starting points.

#### The EM algorithm and discretisation

After finding suitable starting points, STEME runs the EM algorithm on the best starting point for each 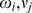 combination. STEME's EM algorithm implementation has been described in some detail in the original STEME paper [14], so we do not repeat it here.

In a similar manner to MEME, STEME performs a discretisation step immediately following the EM algorithm. This discretisation step serves to sharpen the motif and determines the number of sites for which the motif's significance is maximised. For each 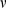 in 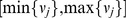 STEME finds the best 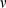 sites in the data for the motif (in the sense that their 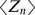 are maximal). STEME builds a motif from these sites and evaluates its significance. The motif with the greatest significance is selected and this is the motif that is reported to the user. In general the discretisation step serves to increase the significance and the information content of the motif.

#### Finding motif instances

At several stages in its algorithm, STEME is required to find the best 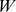-mers in the sequences given a current motif. For example when finding starts, STEME needs the best 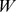-mers given a motif built from the starting point's consensus sequence. In STEME's discretisation step, the best 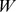-mers given the motif learnt during the EM algorithm must be found. STEME finds these 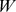-mers using the algorithm described here. Once again, STEME can use the suffix tree representing the input sequences to do this efficiently.

Suppose STEME is looking for the 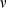 best 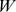-mers. As in the EM algorithm, STEME descends the suffix tree and as it does so it keeps track of an upper bound on the 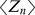 of the 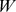-mers represented by the current node. During this descent STEME stores the best 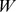-mers seen so far and only descends those parts of the tree which could contribute better 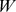-mers. One further detail accelerates this method, STEME descends the suffix tree in an order where preferred bases in the motif are examined first. For example, if ‘A’ was the preferred base at the fifth position in the motif, then STEME would always descend the ‘A’ branches of the suffix tree at depth five first. In this way STEME tends to find the best 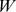-mers earlier and STEME is able to use higher bounds to ignore larger parts of the suffix tree.

Unfortunately in general STEME needs to find non-overlapping 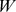-mers, otherwise it converges too easily on motifs that have self-overlapping binding sites. Suffix trees do not lend themselves to analysis of overlaps so STEME removes the overlapping instances as a post-processing step. That is, it looks for the best 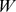-mers without regard for overlaps and when it has found them, only retains the 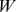-mers that are not overlapped by better 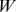-mers.

#### Finding multiple motifs

Like most other motif finders, STEME can find more than one motif concurrently. STEME uses a similar technique as MEME. Once the first motif has been found, its instances are probabilistically erased from the sequences by modifying the position-specific priors. That is, the data in the model is changed to make the instances of the discovered motif less available for binding.

Additionally, we remove any starting points that overlap either the instances of the starting point that was used when finding the motif, or the instances of the motif after the EM algorithm.

#### Significance calculations

In general, the user of a motif finder will not know how many motifs are present in their input sequences. The user will rely on the motif finder to quantify the significance of the motifs discovered. Many different statistics have been used by motif finders to measure significance. STEME uses the same approach as MEME.

STEME assesses a motif's significance using its information content, width and the number of sites it was created from. STEME reports an E-value statistic based on these properties. The E-value represents how many motifs of higher information content we would expect to find in a random set of sequences. Specifically, suppose we drew a hypothetical set of sequences from a 0-order Markov model based on the input sequences. The E-value estimates the expected number of motifs of the same width and number of sites we could find in the random sequences that have a higher information content than the given motif. E-values defined in this way are widely used as measures of motif significance, for example by MEME, Hertz et al. [40] and Nagarajan et al. [22].

Suppose we have a motif built from 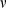 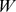-mers. Let 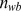 denote the number of occurrences of base 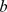 in the 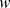
'th position of the motif. The information content of the 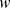
'th position of the motif is defined as
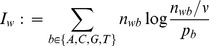
where 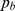 is the probability of base 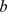 in the background. Due to the independence of the positions of the motif, its information content is
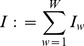
Motifs with higher information content are more interesting as they represent more specific binding preferences. The information content can be used to rank motifs with the same 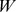 and 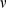. However, the information content does not quantify the significance of the motif directly and does not allow us to compare the significance of motifs of varying 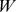 and 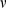. For this we would like to calculate a 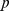-value for the information content. Our null hypothesis is that the 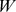-mers that form the motif are drawn from a 0-order Markov model generated from the base pair frequencies in the input sequences. We calculate the probability mass function (p.m.f.) for the information content of a motif built from 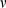 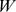-mers drawn at random under this model. An exact calculation of this p.m.f. is intractable for typical 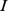, 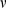 and 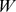 so we use an approximation.

Hirji [41] presented an algorithm to calculate the p.m.f. of the information content of a motif column on a lattice. The same dynamic programming algorithm was later rediscovered by Hertz and Stormo [40]. Nagarajan et al. [22] presented an exponentially shifted version of this algorithm that avoids underflows and hence removes the need for logarithmic arithmetic thus improving the runtime. Nagarajan et al. presented the algorithm as a solution to calculating the p.m.f. of the information content of a column for a fixed 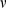. STEME needs to calculate the significance for many different motifs of varying 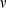. Here we show how Nagarajan's algorithm can be adapted to calculate p.m.f.s for a range of 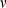 simultaneously.

Extending the notation of section 2.1 in Nagarajan et al. [22] we define
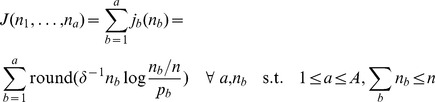
Note we do not assume 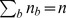 or that 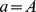 where 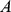 is the size of the alphabet. In the case above where 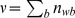 we have
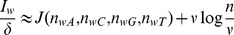
or more precisely
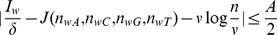
(12)Having established a relationship between 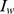 and 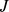 where the accuracy depends on 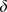, we claim that the 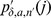 defined in Nagarajan et al. can be interpreted as a p.m.f. for 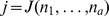 as follows
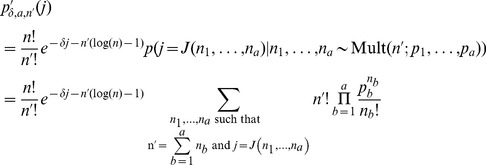
(13)

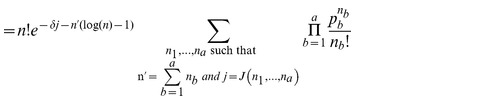
(14)Hence the 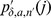 are shifted versions of the 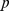-values for the statistics 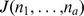 under the null hypothesis. This is a simple generalisation of the claim presented by Nagarajan et al. It has a similarly straight-forward proof by induction that we give here. For 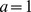, the claim is trivial although we do note a minor correction to Equation (4) presented by Nagarajan et al. We believe the case where 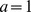 should read
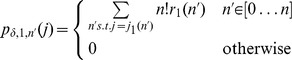
as it is possible for two different 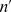 to give the same value of 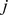. Perhaps this is only possible when 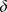 is chosen badly. Continuing our proof by induction, we suppose the claim is true for a given 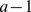 and for all 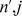 where 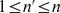. We will show this implies the claim is true for 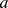.

Re-writing 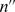 as 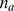 from the shifted version of Equation (4) presented by Nagarajan et al. we have
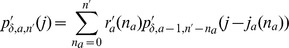
where
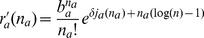
is defined as by Nagarajan et al. Rearranging and using Equation (14), we have
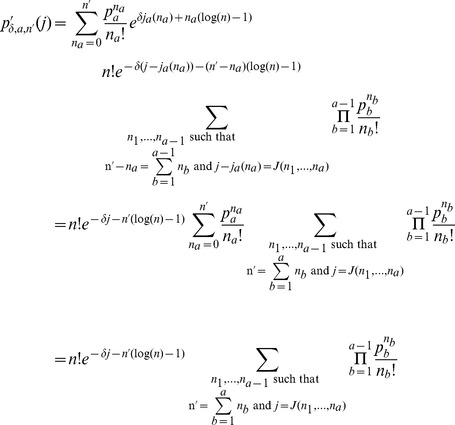
(15)which proves the claim by induction. Equation (13) gives us the p.m.f. of the statistic 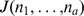 for any 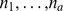 where 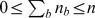 under the null hypothesis. Equation (12) relates 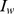 to 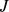 so we have the p.m.f. of 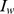 up to the accuracy specified by 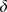.

Once we have the p.m.f. of the information content of a motif column, we use the QFAST algorithm [42] to combine the 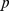-values for all the columns into one 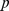-value for the motif. We could have selected any 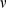 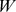-mers from the input sequences but we chose to select 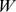-mers to increase the significance of our motif. We need to correct this 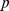-value for the number of different ways we could have chosen 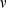 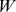-mers from the sequences. Multiplying the 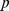-value by this number gives us an E-value, a statistic that we can use to assess the significance of the motif. It is an approximation to the number of motifs we would expect to find with as good or better an information content were we to select the best 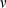 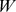-mers from a pool of 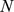 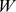-mers distributed as our 0-order Markov model.

#### Position-specific priors

As described in the model section, position-specific priors work at the level of individual 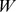-mers. However, as STEME considers several different widths of motifs, STEME will need to consider position-specific priors at all these different widths. We describe here STEME's method for converting position-specific priors from 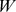-mers to a base pairs and back again.

Suppose STEME found an instance of a motif, 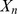, and must probabilistically erase it from the base pair priors, 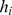. STEME uses the following update rule
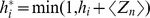
Note that the 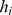 are parameterised in a negative sense so that 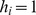 means that the base pair indexed by 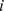 is not available as a binding site. So the availability of all the base pairs in the instance is lowered by 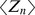.

Conversely, suppose STEME needs position-specific priors for 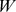-mers. It generates them from the base pair priors as follows
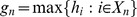
So the prior for 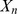 is simply the prior for the least available base pair in 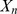.

### Evaluation on mouse ES cell data from Chen et al

The 13 sets of sequences were downloaded from Tim Bailey's website to ensure we used the same data as in his evaluation of DREME. The 5 Kb promoter control sequences were downloaded from UCSC website and repeat masked. We ran DREME (version 4.9.1) with all the default options. STEME (version 1.8.17) was asked to look for motifs of widths, 6, 8, 11, 14 and 18. STEME used a first order Markov background model when using the dinucleotide shuffled control sequences and a fifth order Markov background model when using the promoter control sequences. STEME was asked to search for up to 5 motifs and any motif with a log E-value less than 0 was deemed significant. Trawler (standalone version 1.2) was run with all the default parameters. To compare the recovered motifs to the motif databases we ran the tool TOMTOM (version 4.9.1) from the MEME suite with the same parameters as in Bailey's evaluation: -min-overlap 5 -dist pearson -evalue -thresh 0.05 -no-ssc.
